# Newtonian Equivalence Principles

**DOI:** 10.1007/s10670-021-00513-7

**Published:** 2022-02-13

**Authors:** James Read, Nicholas J. Teh

**Affiliations:** 1 Faculty of Philosophy, University of Oxford, Oxford, UK; 2 Department of Philosophy, University of Notre Dame, South Bend, USA

## Abstract

The equivalence principle has constituted one of the cornerstones of discussions in the foundations of spacetime theories over the past century. However, up to this point the principle has been considered overwhelmingly only within the context of relativistic physics. In this article, we demonstrate that the principle has much broader, super-theoretic significance: to do so, we present a unified framework for understanding the principle in its various guises, applicable to both relativistic and Newtonian contexts. We thereby deepen significantly our understanding of the role played by the equivalence principle in a broad class of spacetime theories.

## Introduction

It is well-established and well-understood that, alongside Mach’s principle and the action-reaction principle, Einstein’s equivalence principle was a critical heuristic device in his path towards general relativity (Lehmkuhl 2021; Norton 1989). What is significantly less well-established, and continues to be the subject of vigorous contemporary research, is the extent to which the equivalence principle plays a role in illuminating the conceptual foundations of general relativity.

Philosophers have played an important role in these investigations. For instance, Lehmkuhl (2021) has proposed that the equivalence principle be understood as a ‘bridge principle’ between general relativity on the one hand, and other spacetime theories—notably Newtonian gravitation and special relativity—on the other. He thus submits that if we are to construct a clear map of the ‘space of spacetime theories’ (Lehmkuhl 2017), it is important to have a clear understanding of the equivalence principle. On the other hand, there is also a long tradition of scepticism about the foundational relevance of the equivalence principle. Synge, for example, declared infamously in 1960 that

The Principle of Equivalence performed the essential office of midwife at the birth of general relativity, but ... I suggest that the midwife be now buried with appropriate honours ... (Synge 1960, pp. ix-x)

To put our cards on the table: we do not share Synge’s negative appraisal of the conceptual import of the equivalence principle;^1^ rather, we believe that the principle has an important role to play both within specific spacetime theories (for reasons to which we will come), and—*à la* Lehmkuhl—as a principle that helps us to understand the relationships between different theories of spacetime.^2^

Of course, vindication of these assertions mandates investigation into how the equivalence principle functions in spacetime theories other than general relativity. One goal of this paper is to pursue such an investigation in the context of a natural analogue of general relativity which has been much discussed by philosophers: Newton-Cartan theory.^3^ What is the status of the equivalence principle in this context, and does it parallel the role of the equivalence principle in general relativity?

In recent years, some preliminary work in this direction has been undertaken: for example, with Newton’s Corollary VI in mind,^4^ Fox (2016), Knox (2014), Saunders (1998, pp. 138–139, 148) and Stachel (2006) all discuss Newtonian versions of the equivalence principle, *qua* unification of gravity and inertia. The topic is also addressed in classic physics works such as (Misner et al. 1973; Thorne et al. 1973). However, it is fair to say that essentially all of this work has focused on the ‘Newtonian equivalence principle’ as articulated within non-geometrized Newtonian gravity, and although e.g.  Knox (2014), Saunders (2013) and Wallace (2020) explore relationships between such principles and Newton-Cartan theory, the fully general connections between general relativistic equivalence principles on the one hand, and intrinsic Newton-Cartan equivalence principles on the other, have yet to be clarified. It is the goal of the present article to undertake exactly this task.

A caveat before we begin: we take ourselves to be working in the tradition of, and indeed at the level of mathematical rigour of, the above-mentioned authors. We recognise that there are issues with rendering mathematically precise some of the versions of the equivalence principle discussed in this paper. To some extent, this is mitigated by our focusing on ‘pointy’ equivalence principles (cf. Ghins and Budden 2001), rather than equivalence principles holding in a neighbourhood. But this does not go all the way to addressing the concerns raised in e.g. (Dewar 2020; Fletcher 2020; Weatherall 2021): that, however, will have to wait for a future piece.

We begin our discussion in the present article by focusing on Knox’s formulation of Newtonian equivalence principles, for she is the only author up to this point (to our knowledge) to discuss explicitly the Newtonian version of the ‘strong equivalence principle’ of general relativity. For our purposes, her discussion is especially telling because of a subtle lacuna in the analysis: she slides between discussing the strong equivalence principle in a geometrized theory of gravitation such as general relativity (where the principle is framed in terms of normal coordinates), to discussing a non-geometrical (or at least: not obviously geometrical) form of the strong equivalence principle in Newtonian gravity (where the principle is framed in terms of a joint symmetry of the flat connection and the gravitational field). This raises an important conceptual puzzle: how should a Newtonian strong equivalence principle be framed in a geometrized theory such as Newton-Cartan theory, and what is the relationship between this formulation and the non-geometrized formulation that Knox gives? Furthermore, a moment’s reflection should push one to wonder whether, perhaps, the analogous question for the relativistic strong equivalence principle is not as well-understood as one might have previously thought. For consider: what is the relationship between the strong equivalence principle in general relativity (the geometrized version) and the strong equivalence principle in the equivalent non-geometrized theory: a theory now known as teleparallel gravity?^5^ In what follows, we will resolve these questions; our analysis will, moreover, reveal a set of links between these resolutions and other recent work in the foundations of spacetime theories—e.g., (Greaves and Wallace 2014; Read et al. 2018; Wallace 2020).

The plan is as follows. In §2, we provide a detailed taxonomy of relativistic equivalence principles, and present the first systematic taxonomy of Newtonian equivalence principles. §2.1 begins with a review of some relevant facts about general relativity and Newton-Cartan theory. §2.2 then lays out a taxonomy of relativistic equivalence principles and shows that, in the relativity literature, there are really two versions of the strong equivalence principle: a geometrized and a non-geometrized version. §2.3 then turns to the non-relativistic scenario, and discusses the Newtonian analogues of these versions of the equivalence principle. Building upon this work, §3 develops in the relativistic case a more incisive approach to understanding the relationship between the geometrized and non-geometrized strong equivalence principles. §3.1 recalls that teleparallel gravity is a non-geometrized theory of relativistic spacetime that is (locally) empirically equivalent to general relativity; for reasons that will become clear in our discussion, it is often called a ‘recovered model’ of general relativity. §3.2 then explains why the non-geometrized strong equivalence principle is really just a way of unpacking the content of the geometrized strong equivalence principle from the perspective of teleparallel gravity. In §4, the strategy of §3 is applied to the non-relativistic setting. §4.1 reviews the relationship between a Newton-Cartan spacetime (the geometric theory) and its ‘recovered’ non-geometric models—*viz*., Newtonian gravitation—using the machinery of teleparallelization. §4.2 then sets about the task of explaining in this non-relativistic context why, once again, these two versions of the strong equivalence principle align; the explanation is exactly parallel to that given in the relativistic case. Drawing upon all this work, in §5 is presented a complete map of equivalence principles, applicable to both the relativistic and Newtonian contexts. §6 concludes.

## Equivalence Principles

In this section, we first recall the relevant details of general relativity and Newton-Cartan theory (§2.1), and of the various important versions of the equivalence principle which arise in the general relativity literature (§2.2). We then present, by analogy with the latter, a taxonomy of Newtonian equivalence principles (§2.3).

### General Relativity and Newton-Cartan Theory

Our interest lies with general relativity and Newton-Cartan theory. Models of the former are triples $\langle M, g_{ab}, T_{ab} \rangle$, where *M* is a differentiable manifold, $g_{ab}$ is a Lorentzian metric field on *M*, and $T_{ab}$ represents the stress-energy content of matter fields. These fields are subject to satisfaction of the Einstein equation

1 $$\begin{aligned} G_{ab} = 8 \pi T_{ab} , \end{aligned}$$

where $G_{ab}$ is the Einstein tensor, plus dynamical equations associated with the matter fields. In general relativity, gravitating but force-free particles follow geodesics of the connection, in the sense that^6^

2 $$\begin{aligned} \xi ^a \nabla _a \xi ^b =0, \end{aligned}$$

where $\xi ^a$ is the velocity vector of the particle under consideration.

Models of Newton-Cartan theory are tuples $\langle M, t_a, h^{ab} , \nabla , \rho \rangle$, where $t_a$ is a 1-form on *M* representing Newtonian absolute time, $h^{ab}$ is a degenerate (inverse) ‘metric’ field on *M* of signature $\left( 0,1,1,1\right)$ satisfying the orthogonality condition $t_{a} h^{ab} = 0$ and representing spatial distance relations instantiated by absolute space, $\nabla$ is a derivative operator satisfying the compatibility conditions $\nabla _a t_{b} = \nabla _a h^{bc} = 0$, and $\rho$ is a scalar matter density field. Models of Newton-Cartan theory are subject to the dynamical equation

3 $$\begin{aligned} R_{ab} = 4\pi \rho t_a t_b, \end{aligned}$$

and the integrability conditions (see e.g. (Malament 2012; Teh 2018) for discussion of the physical significance of these conditions)45Again, in Newton-Cartan theory, gravitating but force-free bodies traverse geodesics of the connection, in the sense that (2) is satisfied.^7^

Given a model of Newton-Cartan theory, one can reconstruct a ‘degeometrized’ model of Newtonian gravity $\langle M, t_a , h^{ab}, {\bar{\nabla }}, \varphi , \rho \rangle$, which now features a different derivative operator ${\bar{\nabla }}$ still satisfying the compatibility conditions, and a real scalar field $\varphi$ representing the gravitational potential, with equations of motion

6 $$\begin{aligned} h^{ab} {\bar{\nabla }}_a {\bar{\nabla }}_b \varphi = 4\pi \rho , \end{aligned}$$

and particle force equation given by

7 $$\begin{aligned} \xi ^a {\bar{\nabla }}_a \xi ^b = - h^{ab} {\bar{\nabla }}_a \varphi , \end{aligned}$$

up to ‘Trautman gauge symmetry’,

8 $$\begin{aligned} {\bar{\nabla }}&\mapsto {\bar{\nabla }}' = \left( {\bar{\nabla }}, t_b t_c {\bar{\nabla }}^a \psi \right) , \end{aligned}$$

9 $$\begin{aligned} \varphi&\mapsto \varphi ' = \varphi + \psi , \end{aligned}$$

where the scalar field $\psi$ must satisfy ${\bar{\nabla }}^a {\bar{\nabla }}^b \psi = 0$. Thus, one finds that an equivalence class of solutions of Newtonian gravity is associated with a single solution of Newton-Cartan theory. Note that models of Newton-Cartan theory do not lie on the Trautman gauge orbits of their associated recovered models;^8^ this observation will be important in our discussions of the equivalence principle.

### Relativistic Equivalence Principles

We begin our presentation of relativistic equivalence principles by recalling three versions of said principle; our taxonomy follows an illuminating recent article by Lehmkuhl (2021), in which are distinguished ‘weak’, ‘Einstein’, and ‘strong’ versions of the principle.^9^ First, we have the weak equivalence principle (Lehmkuhl 2021, p. 4). This comes in two forms:

$$\mathbf{WEP} _{\mathbf{1}}$$
All uncharged test bodies placed at an initial event in spacetime and given an initial velocity follow the same trajectories.^10^
$$\mathbf{WEP} _{\mathbf{2}}$$
For any body, the gravitational mass of that body is equal to its inertial mass.

Regarding $\mathbf{WEP} _{\mathbf{1}}$: we offer a definition of ‘uncharged’ below; by ‘test body’, we mean, following Will, a body “that has negligible self-gravitational energy (as estimated by Newtonian theory) and that is small enough in size so that its coupling to inhomogeneities in external fields can be ignored” (Clifford 2018, p. 16). This is not to say that $\mathbf{WEP} _{\mathbf{1}}$ is thereby expunged of conceptual difficulties—for example, one might still worry that the motions of uncharged test bodies can be functions of their internal constitutions (imagine, for example, a boisterous child on the rear seat of a car). However, in the interests of making progress against our stated goals in this paper, we simply register such concerns and hereby set them aside.^11^ Regarding $\mathbf{WEP} _{\mathbf{2}}$: one could weaken this principle to assert only the proportionality of gravitational and inertial masses. While in this paper we have chosen to use the stronger version of the principle (asserting equality), nothing will hinge upon this decision.

As Lehmkuhl states (2021, p. 4) (building on prior work by Ohanian (1977, p. 904) and Will (2018, p. 16)), $\mathbf{WEP} _{\mathbf{2}}$ implies $\mathbf{WEP} _{\mathbf{1}}$;^12^ it is worth rehearsing the reasoning here. Suppose that $\mathbf{WEP} _{\mathbf{2}}$ holds, and consider a test body with inertial mass $m_I$ and gravitational mass $m_G$ in a gravitational field $G^a$; its force equation is

10 $$\begin{aligned} F^a := m_I \xi ^b \nabla _a \xi ^a = - m_G G^a , \end{aligned}$$

where $\xi ^a$ is the body’s velocity vector. From this, we find that the acceleration of this body is given by $- \frac{m_G}{m_I} G^a$; thus, all such uncharged bodies—the condition of ‘uncharged’ meaning that no further terms beyond that involving $G^a$ can appear on the right-hand side of (10)—located at the same initial spacetime point and given some initial velocity there follow the same trajectories, which is $\mathbf{WEP} _{\mathbf{1}}$.

Given this, a configuration of multiple particles in a uniform gravitational field will preserve the same relative motions among themselves, regardless of the strength of the uniform gravitation field in which they are situated. If one then assumes that such is also the case for uniform inertial accelerations (this being a dynamical assumption about the bodies under consideration, essentially equivalent to Newton’s Corollary VI),^13^ then one recovers what Knox (2014, p. 873), following Stachel (2006), calls the ‘Newstein equivalence principle’ (**NEP**):^14^

**NEP**: No experiment can distinguish between the effects of a homogeneous gravitational field and the inertial effects arising in a uniformly accelerating frame.

Three remarks on **NEP**. First: although presented by both Knox and Stachel in the Newtonian context, it is important to recognize that this principle is not uniquely Newtonian; in this sense, it is also applicable in the relativistic context (hence its inclusion here).^15^ Second: although one might be tempted to identify **NEP** with Newton’s Corollary VI, it is more conceptually careful to regard this principle as being a consequence of Corollary VI, when combined with other salient assumptions.^16^ Third: **NEP** expresses the essential content of Einstein’s rocket thought experiment: everything would look and touch and taste and smell and sound the same in a rocket accelerating upwards at *g* ms^-2^ as in a rocket stationary with respect to the surface of the Earth.^17^ (We return to Einstein’s rocket in §5.)

**NEP** was a crucial starting-point for Einstein in his construction of a new version of the equivalence principle, which Lehmkuhl dubs the ‘Einstein equivalence principle’ (**EEP**). This principle conceptually unifies gravitational and inertial effects—which **NEP** already declares to be empirically indistinguishable (Lehmkuhl 2021, p. 9):^18^

**EEP**: Gravity and inertia are the same in their very essence (‘*wesensgleich*’).

(Here, by ‘gravity’ is meant initially ‘a homogeneous gravitational field’, and by ‘inertia’ is meant initially ‘the effects associated with description with respect to a uniformly accelerating frame’; however, from this one may then take the further conceptual leap of bootstrapping to the unification of gravitational and inertial effects *tout court*—this, indeed, is the leap taken by **EEP**.) On **EEP**, gravitational and inertial effects are conceptually unified, so that the inertial frames of reference (in which there are no inertial effects) just are the freely falling frames. For the purposes of our discussion, it is important to be completely precise on what this statement of **EEP** amounts to mathematically; in the ensuing, we take the above formulation to be synonymous with the following mathematical rendering: (We thus label both ‘**EEP**’.)

**EEP**: Gravitational and inertial effects are conceptually unified, insofar as both are represented by the components of the same (possibly curved) compatible connection.

Thus, our **EEP** already implies a commitment towards a geometrized formulation of the theory under consideration: indeed, in this paper we take whether **EEP** holds in a theory to be definitional of whether that theory is ‘geometrized’. Using **EEP** (and, as mentioned above, other heuristic inputs, such as Mach’s principle), Einstein was, after a long struggle, able to complete his general theory of relativity in 1915.

Finally, we turn to the strong equivalence principle, which is to be articulated within the completed framework of relativistic gravity. Versions of this principle ramify in a number of directions; however, before we present the various options, it is important to recall the following facts about ‘normal coordinates’ on a manifold *M*.

In a coordinate co-frame basis $\left\{ e_\mu \right\}$, the connection components  associated to a derivative operator $\nabla$ are defined by . Then, at any point $p \in M$ we can choose normal coordinates such that  in those coordinates; for a torsion-free derivative operator, we can in fact choose normal coordinates such that . (Note that the connection components away from *p* will in general not vanish.) Taking *M* to be equipped with a Lorentzian metric field $g_{ab}$, if the unique torsion-free, metric compatible derivative operator is used, then in normal coordinates we also have $g_{\mu \nu ,\rho }\left( p\right) =0$, and one can further restrict to orthonormal coordinates at *p* such that $g_{\mu \nu }\left( p\right) = \mathrm {diag}\left( -1,1,1,1\right)$. Since $g_{\mu \nu }\left( p\right)$ takes this diagonal form, one might write $g_{\mu \nu }\left( p\right) = \eta _{\mu \nu }$. This notwithstanding, any claim that the metric field ‘reduces’ to the Minkowski metric at *p* in such coordinates should be met with suspicion—for in general, second (and higher) order derivatives of the metric field do not vanish at *p*, in these coordinates.

With these facts in mind, we can now present various versions of the strong equivalence principle. There are two different possible forms of this principle, depending on whether **EEP** is endorsed. First assuming **EEP**, the principle reads as follows:

$$\mathbf{SEP}_{{\mathbf{1, EEP}}}$$:
At any $p \in M$, one can find an orthonormal normal frame in which gravito-inertial effects, as represented by connection coefficients, vanish.

Note that the status of $\mathbf{SEP} _{\mathbf{1}}$ is different if one does not assume **EEP**: in this case, the principle states not that one can find a coordinate system such that connection coefficients vanish, but rather that one can find a coordinate system such that connection coefficients cancel gravitational effects, as represented by some tensor quantity (cf. Knox 2014, p. 874).

Thus, explicitly, one has:^19^

At any $p \in M$, one can find a frame in which inertial effects, as represented by connection coefficients, cancel gravitational effects, as represented by some tensor quantity.

(In , by ‘cancellation’, we of course mean cancellation of the components of the objects in question in some coordinate system.)

Sometimes, $\mathbf{WEP} _{\mathbf{1}}$ is supplemented with a statement about the classes of coordinate frames in which the principle obtains, thereby leading to (two versions of) what we dub $\mathbf{SEP} _{\mathbf{2}}$. In the relativistic framework, these two versions of $\mathbf{SEP} _{\mathbf{2}}$ read as follows:

$$\mathbf{SEP}_{{\mathbf{2, EEP}}}$$:
$\mathbf{SEP}_{{\mathbf{1, EEP}}}$ holds, and the frames in which this principle holds are related by Lorentz transformations.
holds, and the frames in which this principle holds are related by Lorentz transformations.

When one has the geometrical construction of normal coordinates in mind, in the case of Lorentzian manifolds, the Lorentz symmetries in $\mathbf{SEP} _{\mathbf{2}}$ are inherited from the Killing symmetries (that preserve points) of the tangent space via the standard ‘exponential map’ construction of those coordinates.^20^

There are three further important remarks which we must make on all the above versions of the strong equivalence principle. First: we have chosen to focus on what Ghins and Budden refer to as ‘pointy’ versions of the strong equivalence principle (Ghins and Budden 2001), in order to avoid delicate issues of approximation which enter the fold when one seeks to extend to ‘neighbourhood’ versions of the strong equivalence principle: although we do think that such extensions can be made (see Read et al. 2018 for details which we endorse), these are not our concern in this paper. Second: often, presentations of the strong equivalence principle make reference to the frames in which the dynamical equations governing non-gravitational fields take their simplest form (see e.g. Knox 2013; Read et al. 2018). Our above versions of the principle are compatible with such statements: the frames in which the dynamical equations take their simplest form may be understood to be the frames in which (in the geometrized framework, say) connection coefficients can be made to vanish. Note that on this dynamical understanding, very little is guaranteed *ab initio*—in particular, it is not secured from the outset that all dynamical equations will take their simplest forms in the same frames of reference;^21^ moreover, it is not guaranteed that these frames are those in which the presentation of the geometrical structure of the theory simplifies maximally. These are thus to be understood as two additional input assumptions, or ‘miracles’, in any particular spacetime theory—see (Read et al. 2018) for detailed discussion. Third (and relatedly): in Read et al. (2018), the issue of second-order equations leading to the appearance of curvature terms in dynamical equations, even at a point $p \in M$, is discussed. Again, while this is an interesting issue, it will not bear upon any of our constructions in this paper.^22^

Thus, to summarize, we have a tolerably clear web (albeit not a straightforward hierarchy) of equivalence principles. $\mathbf{WEP} _{\mathbf{2}}$ implies $\mathbf{WEP} _{\mathbf{1}}$, which in turn implies **NEP** (when combined with certain extra assumptions regarding the dynamics of the matter under consideration, in particular assumptions akin to Newton’s Corollary VI). **EEP** provides a conceptual unification of gravity and inertia. $\mathbf{SEP} _{\mathbf{1}}$ regards the local transforming away of gravitational/gravito-inertial effects; this can be read in two different ways, depending upon whether **EEP** is also endorsed. $\mathbf{SEP} _{\mathbf{2}}$ regards the transformations which relate such local coordinate systems.

With this web of equivalence principles in hand, we turn now to Newtonian equivalence principles; we will see that an analogous pattern of principles arises in that context. Once the connections between these different versions of the equivalence principle (in both the relativistic and Newtonian contexts) have been articulated in more detail, we will present the links diagrammatically in §5.

### Newtonian Equivalence Principles

There is nothing relativistic about $\mathbf{WEP} _{\mathbf{1}}$, $\mathbf{WEP} _{\mathbf{2}}$, and **NEP**: the possibility of their holding applies in the Newtonian context just as much as in a relativistic framework. (In a sense, of course, this is not surprising, since all three of these principles have their origins in pre-relativistic physics!) Similarly, **EEP** is a conceptual unification of gravitational and inertial effects; it applies equally well in the Newtonian as in the relativistic context. (Indeed, there is a sense in which Trautman geometrization affords the precise mathematical expression of **EEP** in the Newtonian case; as we will see later, the analogous mathematical expression of **EEP** in the relativistic case would be the geometrization of a teleparallel theory.) The only modification necessary is that ‘compatible’ in the Newtonian context means: compatible with $t_{a}$ and $h^{ab}$.

Thus, it is only at the level of the strong equivalence principle that there can be any difference between the relativistic and Newtonian equivalence principles. Unsurprisingly, Knox dubs the Newtonian version of this principle the ‘Newtonian strong equivalence principle’ (Knox 2014, p. 874). From context, it is clear that by this she has in mind —i.e., the cancelling of gravitational effects in Newtonian gravity on making a judicious choice of frame.^23^ Our first point to make here is that it is also possible to articulate $\mathbf{SEP}_{{\mathbf{1, EEP}}}$ (i.e., the geometrized version of the strong equivalence principle) in the Newtonian case: this is simply a statement of the existence of normal coordinates in Newton-Cartan theory—a statement which is *true*, in light of the fact that the Newton-Cartan derivative operator is torsion-free.^24^^,^^25^ What are the transformations relating these normal coordinate systems? As before, we note that, from a geometrical point of view, by the standard ‘exponential map’ construction of normal coordinates, this set will inherit the Killing symmetries that stabilize a point of the tangent space. In our present Newton-Cartan setting, the tangent space Killing symmetries are the Galilean transformations—see (Duval 1993). Knox does not extend her Newtonian version of the strong equivalence principle to apply also to the symmetries of the spacetime structure, but in light of this result, it is clear how to do so:

$$\mathbf{SEP}_{{\mathbf{2, EEP}}}$$:
$\mathbf{SEP}_{{\mathbf{1, EEP}}}$ holds, and the frames in which this principle holds are related by Galilean transformations.
holds, and the frames in which this principle holds are related by Galilean transformations.

Thus, up to the specific structure of the symmetry group in $\mathbf{SEP} _{\mathbf{2}}$, we have exactly the same web of equivalence principles in the Newtonian case as in the relativistic context. With this established, in the following two sections we consider in greater detail the mathematical details of these equivalence principles, and their interrelations.

## Relativistic Equivalence Principles

In this section, we turn to the mathematical details of the above taxonomy of equivalence principles in the relativistic context. We begin in §3.1 by recalling the connections between general relativity and teleparallel gravity. We then consider relativistic equivalence principles in §3.2. We undertake the parallel tasks for Newtonian theories in §4.

### Relativistic Recovery

To present teleparallel gravity (which, recall, is a ‘degeometrized’ relativistic theory, which is locally empirically equivalent to general relativity, but in which gravity acts as a force: see (Aldrovandi and Pereira 2013) for relevant background, and (Knox 2011; Read 2016; Read and Teh 2018; Wallace 2015) for philosophical discussion^26^), we must first define a ‘vielbein’. When working with Lorentzian manifolds (as in general relativity), at each $p \in M$ we can always find objects  such that11With this in mind, let a vielbein  be a map from tangent spaces to fibres of a Minkowski vector bundle ${\mathcal {M}} M$; the  are inverse vielbeins, such that . Note that there is a gauge freedom here: a Lorentz transformation of the internal indices of the vielbein would yield the same metric field $g_{ab}$ (see e.g. Aldrovandi et al. 2003, p. 550).

Given a vielbein, we define the components  of a ‘Weitzenböck derivative operator’ ${\bar{\nabla }}$ to be:^27^12${\bar{\nabla }}$ is the derivative operator of teleparallel gravity. This in hand, we declare that models of teleparallel gravity are triples , where  is a vielbein on *M*, and, as in general relativity, $T_{ab}$ represents the stress-energy content associated with matter fields. Analogously with general relativity (and in contrast with Newtonian gravity and Newton-Cartan theory), the Weitzenböck derivative operator ${\bar{\nabla }}$ is not included as a separate element in the model, for, given a vielbein , there exists a unique such operator, defined via (12). Dynamically, solutions of vacuum teleparallel gravity satisfy the field equation^28^13where  with $g_{\mu \nu }$ defined as in (11) (Aldrovandi and Pereira 2013, p. 5), and the teleparallel gravitational current, superpotential, and Lagrangian are respectively given by:In the above,  is the ‘torsion tensor’, which encodes the antisymmetric part of the connection; the ‘contorsion tensor’  is defined as the difference between the Weitzenböck and Levi-Civita connection components. Note that, unlike the Levi-Civita derivative operator $\nabla$ of general relativity, the torsion of the Weitzenböck derivative operator ${\bar{\nabla }}$ of teleparallel gravity does not necessarily vanish. In teleparallel gravity, gravitating but otherwise force-free test particles satisfy

14 $$\begin{aligned} \xi ^a {\bar{\nabla }}_a \xi _b = \xi ^c {\bar{T}}_{cba}\xi ^a . \end{aligned}$$

Thus, teleparallel gravity can be understood to be a force theory empirically equivalent to general relativity (in several senses—including that Eq. (16) of teleparallel gravity can be derived from the geodesic equation of general relativity (2), and vice versa—see e.g. Aldrovandi and Pereira 2013; Knox 2011; Read 2016 for details), just as Newtonian gravity is a force theory empirically equivalent to Newton-Cartan theory.^29^

### Refined Relativistic Equivalence Principles

The above in hand, it is straightforward to articulate precisely $\mathbf{SEP}_{{\mathbf{1, EEP}}}$, $\mathbf{SEP}_{{\mathbf{2, EEP}}}$, , and  in the relativistic setting. Beginning with the former principle, this follows simply in virtue of the fact that, in general relativity, at any $p \in M$ it is always possible to find a normal coordinate system in which the Levi-Civita connection coefficients  vanish. Writing the geodesic Eq. (2) in a coordinate basis,15we see that, in such normal coordinate systems, test particles follow flat spacetime straight-line trajectories. As already discussed, the coordinate transformations relating these normal frames are inherited via the exponential map from the Killing symmetries of the tangent space—i.e., are the Lorentz transformations; this suffices to recover $\mathbf{SEP}_{{\mathbf{2, EEP}}}$.

On the other hand, consider now . Writing the teleparallel gravity force equation Eq. (16) in a coordinate basis, we have16Choosing  would amount to choosing a frame (not normal with respect to the Weitzenböck connection) such that inertial effects cancel the universal gravitational force (as given by torsion, in teleparallel gravity); this is . Now using that the contortion tensor  is the difference between the Levi-Civita and Weitzenböck connection components, and the result (Aldrovandi et al. 2003, p. 552)17we see that this choice amounts exactly to setting the Levi-Civita connection coefficients  to vanish (for further details on this calculations, see Aldrovandi and Pereira 2013, p. 65). But we have already seen above that the Levi-Civita normal coordinate systems in which the  vanish are related by Lorentz transformations—so we know (via geometrization of the recovered teleparallel theory) that the coordinate systems in teleparallel gravity in which inertial effects are selected so as to cancel gravitational effects are related by Lorentz transformations; this, then, is .

Note that, in this case, the internal Lorentz gauge symmetries of the recovered teleparallel model (discussed in §3.1) coincide with the transformations which relate the frames in which the strong equivalence principle (in either its geometrized or ungeometrized form) holds. This is no surprise, since in both cases these are inherited from the Killing symmetries of the tangent space. When it comes to the case of the Newtonian equivalence principle discussed below, the situation in this regard will be more subtle, for reasons relating to the fact that, in that case, one must work with an ‘extended’ vielbein formalism.

## Newtonian Equivalence Principles

Our purpose in this section is to show that a complete understanding of Newtonian equivalence principles can be achieved by treating the situation in this context as exactly parallel to the relativistic case. In order to do so, we first need to show, following (Read and Teh 2018), the sense in which Trautman recovery just is a case of teleparallelization; we survey the relevant details in §4.1. We then return to Newtonian equivalence principles in §4.2.

### Non-Relativistic Recovery

This section has a simple take-home: Newtonian gravity is the teleparallel equivalent of Newton-Cartan theory; the relationship between these theories is exactly parallel to that between teleparallel gravity and general relativity. While the torsion in teleparallel gravity is spacetime torsion, the torsion in Newtonian gravity (associated with $\nabla _a \varphi$, as appearing in the force Eq. (7)) is ‘internal’, and associated (via the Cartan equations) with a ‘mass gauge field’ $m_a$, which is a component of an extended vielbein. The remainder of this section seeks to spell out these results in greater detail; readers unconcerned with the technicalities may skip to §4.2.^30^

In order to demonstrate that Newton-Cartan theory stands to Newtonian gravity exactly as general relativity stands to teleparallel gravity, it is first necessary to write Newton-Cartan theory in the vielbein formalism.^31^ In this theory, an extended vielbein is a one-form ; it is ‘extended’ in the sense that it includes the piece $m_a$, needed to parameterize the freedom to change the Lagrangian of the point particle under consideration coupled to a Newton-Cartan background;^32^ in line with this extension, we introduce the five-dimensional ‘internal’ index *I*. The associated (extended) spin connection  consists of the ‘rotation connection’  and the ‘boost connection’ .^33^ It is straightforward to define the standard objects of Newton-Cartan theory in terms of the objects of the theory in its vielbein formulation: , and $t_a$ appears directly in the vielbein. In order to discuss certain frame-dependent quantities, it is convenient to introduce the notion of a ‘observer vector field’—*viz*., a vector field $n^a$ that is timelike and normalized ($t_a n^a = 1$).

We can use the vielbein and the spin connection to equip Newton-Cartan spacetime with a compatible spacetime connection $\nabla$, by means of the vielbein hypothesis . Explicitly, $\nabla$ has the Christoffel symbols^34^18where $n^a$ is an observer vector field,19and

20 $$\begin{aligned} ({\varvec{F}})_{ab} :=({\varvec{\omega }}_i \wedge {\varvec{e}}^i)_{ab} \end{aligned}$$

is the ‘Newton-Coriolis 2-form’.^35^ (18) shows us that, unlike Lorentzian spacetime, Newton-Cartan spacetime does not induce a unique compatible torsion-free spacetime connnection. Instead, the space of possible connections can be parametrized by fixing an observer field $n^a$ which determines , and then further specifying  in order to pick out . These two pieces of data have an important physical interpretation:  is an ‘inertial connection’ in the sense that $n^a$ is acceleration-free and vorticity-free with respect to , and , where $\alpha ^a := n^b \nabla _b n^a$ is the ‘acceleration’ and  is the ‘vorticity’ of $n^a$ with respect to . Finally, it is useful to note that the spacetime boost connection  can be written as (cf. Geracie et al. 2015)21where  is the ‘expansion’ of $n^a$.

Using the Cartan equations (see e.g. Wallace 2015), we can compute the ‘internal’ curvatures associated with the two components of the Newtonian spin connection, i.e. the boost connection ${\varvec{\omega }}^i$ and rotation connection ${\varvec{\omega }}^{ij}$. These are, respectively (cf. Andringa et al. 2011),

22 $$\begin{aligned} {\varvec{R}}^i\left( G\right)&=d{\varvec{\omega }}^i-{\varvec{\omega }}^{ij}\wedge {\varvec{\omega }}_j , \end{aligned}$$

23 $$\begin{aligned} {\varvec{R}}^{ij}\left( J\right)&=d{\varvec{\omega }}^{ij} . \end{aligned}$$

Here, *G* and *J* refer, respectively, to generators of Galilean boosts and spatial rotations, to which the rotation and boost connections are associated. In addition to these curvatures, we can compute the ‘internal’ torsions associated with each of the components of the Newtonian extended vielbein, again using Cartan’s equations. These are:

24 $$\begin{aligned} {\varvec{T}}\left( H\right)&= d{\varvec{t}}, \end{aligned}$$

25 $$\begin{aligned} {\varvec{T}}\left( P\right)&= d{\varvec{e}}^i - {\varvec{\omega }}^{ij}\wedge e_j - {\varvec{\omega }}^i \wedge {\varvec{t}} , \end{aligned}$$

26 $$\begin{aligned} {\varvec{T}}\left( M\right)&= d{\varvec{m}}-{\varvec{\omega }}^i\wedge {\varvec{e}}_i . \end{aligned}$$

Here, ${\varvec{T}}\left( H\right)$, ${\varvec{T}}\left( P\right)$ and ${\varvec{T}}\left( M\right)$ are, respectively, associated with the extended vielbein components ${\varvec{t}}$, ${\varvec{e}}^i$ and ${\varvec{m}}$; *H*, *P* and *M* refer, respectively, to the generators of temporal, spatial and mass translations, to which these vielbein components are associated. Together, $\left\{ G,J,H,P,M\right\}$ are the generators of the ‘Bargmann group’, which is the central extension of the Galilean group.

Since the spacetime connection $\nabla$ of Newton-Cartan theory is torsion-free, we set the torsions associated with ${\varvec{t}}$ and ${\varvec{e}}^i$ to zero. Indeed, by analogy with the torsion-freeness of general relativity, we require that the mass torsion of Newton-Cartan theory also vanish. Thus, we have ${\varvec{T}}\left( H\right) = {\varvec{T}}\left( P\right) = {\varvec{T}}\left( M\right) = 0$. From the condition that the mass torsion vanish, it follows that we have $d{\varvec{\hat{m}}} = {\varvec{\hat{\omega }}}_a \wedge {\varvec{\hat{e}}}^a$. (Here, we include hats to indicate that we are dealing with objects in Newton-Cartan theory, in which all torsions have been set to vanish.) From these conditions, one can determine the spin connection solely in terms of the extended vielbein, thus determining the Newton-Cartan spacetime connection $\nabla$.

We now introduce teleparallel Newton-Cartan theory by identifying the data  that yields (i) a flat spacetime connection ${\bar{\nabla }}$ (the analog of the Weitzenböck connection of teleparallel gravity), and (ii) whose Cartan torsion compensates for the curvature of $\nabla$ (the analogue of the Levi-Civita connection of general relativity). While one might be tempted to introduce non-trivial spatial torsion, we note that for a flat connection, the Bianchi identities imply that this must vanish in the Newtonian case, so it is only the mass torsion which is relevant here.

First, we perform a preliminary ‘inertial gauge-fixing’ that is motivated by (18) and the ensuing discussion: recall that  determines the inertial connection  precisely when it is subject to the constraint ${\varvec{F}} =0$, upon which ${\varvec{T}}\left( M\right) =d{\varvec{m}}$ (because the final term in (26) just is the Newton-Coriolis 2-form). Implementing this constraint and setting ${\varvec{\hat{m}}}={\varvec{m}}$ then yields the following relationship between the Newton-Cartan and teleparallel-equivalent equations of motion:27The above gauge-fixing ${\varvec{F}}=0$ is not invariant under local Galilean boosts. We can remedy this by transforming a vielbein to the ‘twistless gauge’ by means of ${\varvec{e}}^i \mapsto {\varvec{{\bar{e}}}}^i= {\varvec{e}}^i - m^i {\varvec{\tau }}$, where $m^i := e^{ai} m_a$, resulting in the new extended vielbein ${\varvec{{\bar{e}}}}^I= \left( {\varvec{\tau }} ,{\varvec{{\bar{e}}}}^i, {\varvec{\tau }} \varphi \right)$, where $\varphi$ is a boost-invariant scalar. The corresponding frame contains a boost-invariant observer field . This choice is invariant under local boosts acting on the unbarred quantities; however, there are still residual mass gauge symmetry transformations ${\varvec{m}} \mapsto {\varvec{m}} + df$ (where *f* is an arbitrary smooth function), under which $(z^a, \varphi )$ is transformed into another boost-invariant pair $(z'^a, \varphi ')$.

The name ‘twistless gauge’ stems from the observation that the vorticity of $z^a$ vanishes with respect to $\nabla$, so our frame is twistless with respect to the Newton-Cartan connection. In the twistless gauge, it follows immediately from (27) that

28 $$\begin{aligned} \xi ^a \nabla _a \xi ^b = 0 \quad \Longleftrightarrow \quad \xi ^a {\bar{\nabla }}_a \xi ^b = - h^{ba} (d\varphi )_a, \end{aligned}$$

thus recovering the Newtonian gravity force law as the teleparallel equation of motion.

Having gauge-fixed to the inertial connection , we see from (21) that there is only one parameter left to gauge-fix in , *viz*. the expansion . We do so by setting , which along with the ${\varvec{R}}^{ij}\left( J\right) =0$ condition implies that  is flat. One can then deduce easily that the Newton-Cartan source equation holds just in case the flat teleparallel connection $\overset{z}{\nabla }$ satisfies the Newtonian Poisson equation. This absorbs the Trautman recovery theorem into the machinery of Newtonian teleparallelization with appropriate gauge-fixing, up to the standard Trautman gauge symmetry embodied in (8) and (9). In fact, this symmetry is accounted for by the fact that the data  satisfying the twistless gauge and  is unique only up to mass gauge transformations that preserve these conditions.

A final word on the generality of these results. The teleparallelization procedure presupposes that the manifold be parallelizable (i.e., that the tangent bundle be a trivial bundle). While this is necessary in Newton-Cartan theory if one seeks global solutions, it is not necessary in general relativity. This means that the procedure is more general in the Newtonian context than in the relativistic context, insofar as the possibility of conversion into a force theory arises for all solutions of Newton-Cartan theory, but only for a proper subset of solutions of general relativity. In turn, one might argue thereby that the spectre of conventionality of geometry looms larger in the former than in the latter context. For further related discussion of conventionality in these contexts, see (Dürr 2021; Knox 2014; Weatherall and Manchak 2014).

### Refined Newtonian Equivalence Principles

In light of the foregoing, Newtonian gravity is the teleparallel equivalent of Newton-Cartan theory; the relationship between these theories is exactly parallel to that in the relativistic case, and this subsumes and generalizes Trautman recovery. With this in hand, an avenue should be opened up to a more rigorous and systematic understanding of Newtonian equivalence principles, which mirrors the results in the relativistic case presented in §3.2.

Once again, we seek to articulate precisely $\mathbf{SEP}_{{\mathbf{1, EEP}}}$, $\mathbf{SEP}_{{\mathbf{2, EEP}}}$, , and , this time in the Newtonian setting. Beginning with the former, one can again write the particle equation of motion in a coordinate system as per (15), from which $\mathbf{SEP}_{{\mathbf{1, EEP}}}$ follows on setting the Newton-Cartan connection coefficients to vanish. In this case, the coordinate transformations which relate these normal frames are inherited via the exponential map from the Killing symmetries of flat Newton-Cartan spacetime—these are the Galilean transformations (Duval 1993); this thus yields $\mathbf{SEP}_{{\mathbf{2, EEP}}}$.

Now consider . Writing the Newtonian gravity force Eq. (7) in a coordinate basis and recalling that $\xi ^a$ is timelike and normalized, we have29As in the relativistic case, choosing  would amount to choosing a frame (not normal with respect to the Newtonian gravity equivalent of the Weitzenböck connection) such that inertial effects cancel the universal gravitational force (now given by $\nabla _a \varphi$; in the teleparallel interpretation of Newtonian gravity, as we have seen, this is associated with mass torsion); given (8), we see that this choice amounts exactly to setting the Newton-Cartan connection coefficients  to vanish. We have already seen above, however, that these Newton-Cartan normal coordinate systems are related by Galilean symmetries—so we know (via geometrization) that the coordinate systems in which inertial effects are selected to cancel gravitational effects are related by Galilean transformations; this is .

Note that, since Newtonian teleparallelization involves working in the extended vielbein formalism involving the mass gauge field $m_a$, the Killing symmetries of flat Newton-Cartan spacetime (the Galilean transformations) do not in this case coincide with the (Trautman) gauge symmetry of the recovered models, as encapsulated in (8) and (9). However, after stipulating that the gravitational potential $\varphi$ can change only by constant shifts, so that $\nabla ^a \psi = 0$ in (8) (typically such shifts are regarded as being unphysical^36^), the residual Trautman symmetry precisely corresponds to the familiar rigid Galilean transformations.

## Map of Equivalence Principles

**Fig. 1:**
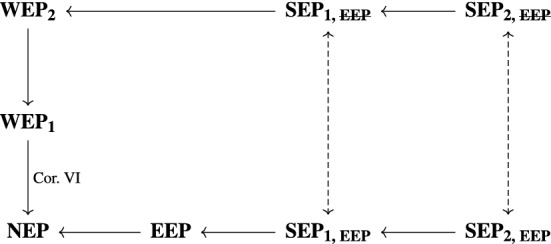
Map of equivalence principles. Solid arrows are logical implication within a theory; dashed arrows are inter-theoretic relations (given by geometrization and recovery)

We have presented a web of equivalence principles which, in light of the fact that Newtonian gravity is the teleparallel equivalent of Newton-Cartan theory, holds equally well in the Newtonian case as in the relativistic case. Given this, we are now in a position to present the complete map of these equivalence principles—see Fig. 1. Of course, as our discussion of $\mathbf{WEP} _{\mathbf{1}}$ in §2.2 should indicate already, we make no claim that all conceptual issues regarding the versions of the equivalence principle discussed in this paper are thereby resolved; this notwithstanding, we regard the links presented in Fig. 1 as a fruitful means of understanding how such principles relate to one another in both relativistic and Newtonian contexts.

Let us review the links on this map. We have already seen in §2.2 (following Lehmkuhl 2021) that $\mathbf{WEP} _{\mathbf{2}}$ implies $\mathbf{WEP} _{\mathbf{1}}$; we have also claimed that, with additional input assumptions akin to Newton’s Corollary VI (hence the note ‘Cor. VI’ in Fig.  1), $\mathbf{WEP} _{\mathbf{1}}$ implies **NEP**. So far, so simple.

Next, **EEP** implies **NEP**, in the sense that if gravity and inertia are conceptually unified, then of course they are empirically indistinguishable. This being said, there is clearly also a sense in which this implication does not hold, for **NEP** trades in terms such as ‘gravity’ and ‘inertia’, which arguably do not make sense in a geometrical context. Moreover, even given the conceptual unification of gravity and inertia as per **EEP**, one can still, within the context of a geometrized theory, effect an arbitrary gravity/inertia split (cf. Lehmkuhl 2014, where Einstein’s thinking of these matters by analogy with the unification of the electric and magnetic fields in Maxwell’s electromagnetism is discussed in detail); relative to that split, the notions can become meaningful—but one would not want to say that, relative to such a split, the notions of gravity and inertia are necesarily empirically indistinguishable (cf. again the case of electromagnetism: relative to a frame, one has well-defined $\mathbf {E}$ and $\mathbf {B}$ fields, but it is not necessarily the case that those fields are empirically indistinguishable). While all of these points are important to register, we also maintain that there is a straightforward sense (articulated above) in which this implication does hold; it is in this sense which this implication in Fig. 1 is intended.

We turn next to implications regarding strong equivalence principles. Clearly, $\mathbf{SEP}_{{\mathbf{1, EEP}}}$ implies **EEP**, for the former is a strictly logically stronger statement. The bidirectional arrows relating the two forms of the geometrized and ungeometrized strong equivalence principle hold in light of geometrization/recovery (to be understood in the unified framework of teleparallelization);^37^ clearly, being strictly logically stronger, both versions of $\mathbf{SEP} _{\mathbf{2}}$ imply their counterpart versions of $\mathbf{SEP} _{\mathbf{1}}$. (We have dotted the arrows associated with geometrization/recovery in order to indicate that they are inter-theoretic, rather than intra-theoretic, relations—in contrast with the other links on this diagram.) This just leaves one link outstanding: that from  to $\mathbf{WEP} _{\mathbf{2}}$. This is not a link which we have discussed up to this point, but it is one which is straightforward to establish—and, indeed, it is one which has already been considered by Weatherall (2011, p. 432). The essential insight is that, working with a degeometrized theory (hence ), multiplying through the recovered force equation ((7) in the Newtonian case) by inertial mass yields immediately the identity of gravitational and inertial mass—i.e., $\mathbf{WEP} _{\mathbf{2}}$. Thus, the final link in Fig. 1 is secured.

It is worth pausing a little longer on this result from Weatherall. He claims to derive $\mathbf{WEP} _{\mathbf{2}}$ in Newtonian gravity, via a two-step process: (i) taking a Newtonian limit of general relativity, to arrive at Newton-Cartan theory (using a version of Ehlers’ ‘frame theory’—see Ehlers 1991; Fletcher 2019; Malament 1986), and (ii) degeometrizing Newton-Cartan theory via Trautman recovery, to arrive at the particle force Eq. (7) of a recovered model. On obtaining this latter equation, the coincidence of gravitational and inertial masses is immediate, as already discussed. In our terminology of equivalence principles, this is a case of (a) beginning with $\mathbf{SEP}_{{\mathbf{1, EEP}}}$ in the relativistic case, (b) moving to the same principle in the Newtonian case by taking the Newtonian limit, (c) d in the Newtonian context, and (d) deriving the Newtonian $\mathbf{WEP} _{\mathbf{2}}$ as a consequence of this. These steps involve following a clear path through Fig. 1; thus, the web of links which we have constructed between equivalence principles is able to subsume and contextualize Weatherall’s result.

Two further comments on this matter. First: given that degeometrization commutes with taking the Newtonian limit (at least in certain cases),^38^ there is another route to Weatherall’s result: (i) degeometrize general relativity, to obtain teleparallel gravity (in which, by direct analogy with Weatherall’s reasoning, one may already show that inertial and gravitational masses coincide, even in a relativistic setting), and (ii) take the Newtonian limit, to obtain Weatherall’s result in Newtonian gravity.

Our second comment is the following: Weatherall’s derivation of $\mathbf{WEP} _{\mathbf{2}}$ is a case of an inter-theoretic derivation of an equivalence principle. This is to be contrasted with an intra-theoretic derivation of an equivalence principle. Two examples of this are the following. First, Greaves and Wallace (2014, §8), consider a subsystem-environment decomposition in Newtonian gravity, and are able to derive the following:

The prediction ... is that in Newtonian gravity, a system floating freely in space behaves identically, with respect to its internal processes, to a system freely falling in a uniform gravitational field. The empirical symmetry associated to [this] is Einstein’s elevator, the thought experiment that led Einstein to the equivalence principle. (Greaves and Wallace 2014, p. 77)

That is, Greaves and Wallace are able to derive **NEP**, within Newtonian mechanics. A second example of an intra-theoretic derivation of an equivalence principle is the following: Wallace (2020) shows that, beginning with a Newton-Cartan model, imposition of appropriate boundary conditions on a subsystem leads to emergent Galilean symmetries of that subsystem. This can be understood to be a derivation of the finite subsystem analog of  within a geometrized theory, because upon taking the far-zone limit in which the subsystem (mass and size) shrinks down to a point within the environment spacetime, an inertial frame (in which the emergent laws governing the subsystem simplify maximally) becomes a normal frame, and one obtains our pointwise construction. (In spite of our focus on pointwise constructions, we concur with Wallace’s plea that the equivalence and relativity principles be considered not merely in the pointwise sense (Wallace 2017). We defer to a future article a full analysis of the relationship between asymptotic, finite, and pointwise constructions.)

## Conclusions

We are now in a position to revisit Lehmkuhl’s claims regarding the role of the equivalence principle as a ‘bridge principle’, linking general relativity to both special relativity (as with $\mathbf{SEP}_{{\mathbf{2, EEP}}}$), and to Newtonian mechanics (as with **EEP**) (Lehmkuhl 2021). Both of these claims are correct, but can be generalized. On the former: Lehmkuhl has identified the role of $\mathbf{SEP}_{{\mathbf{2, EEP}}}$, insofar as it recovers locally in general relativity the symmetries of special relativistic spacetime structure (cf. Read et al. 2018). However, generalizing beyond the relativistic context, one can see that the role of $\mathbf{SEP}_{{\mathbf{2, EEP}}}$ is to link a geometrized theory locally to the flat spacetime tangent space symmetries of that theory; this affords a more general understanding of the role of this principle. Thus, the role of $\mathbf{SEP}_{{\mathbf{2, EEP}}}$ in the context of Newton-Cartan theory is to recover locally the Galilean spacetime symmetries. On **EEP**, we have seen above that this principle expresses a commitment to a geometrized spacetime setting. In that sense, however, Lehmkuhl’s statement that **EEP** links general relativity on the one hand, and Newtonian gravity on the other, is not entirely natural: rather, within the relativistic framework, **EEP** is best understood as linking general relativity and teleparallel gravity; Newtonian gravity, with its own gravity-inertia split, can then be recovered by taking the Newtonian limit.

## The Mass Gauge Field

The purpose of this appendix is to explain in greater detail the physical meaning of the mass gauge field $m_a$ in the vielbein formulation of Newtonian theories. The commutation relations of the Bargmann algebra (i.e., the commutation relations satisfied by the generators of the Bargmann group) are:

30 $$\begin{aligned} \left[ J_{ij} , J_{kl} \right]&= 4 \delta _{[i[k} J_{l]j]} , \end{aligned}$$

31 $$\begin{aligned} \left[ J_{ij} , P_k \right]&= -2 \delta _{k[i}P_{j]} , \end{aligned}$$

32 $$\begin{aligned} \left[ J_{ij} , G_k \right]&= -2 \delta _{k[i} G_{j]} , \end{aligned}$$

33 $$\begin{aligned} \left[ G_i, H \right]&= -P_i , \end{aligned}$$

34 $$\begin{aligned} \left[ G_i , P_j \right]&= -\delta _{ij} M. \end{aligned}$$

When $M=0$, these are the commutation relations associated with the Galilean algebra. Why, then, should we consider $M \ne 0$? The answer to this question can be motivated by considering the action for a free, non-relativistic particle with mass *M*:^39^

35 $$\begin{aligned} S = \frac{1}{2}\int _{t_1}^{t_2} M {\dot{x}}^i {\dot{x}}^i dt . \end{aligned}$$

Under a Galilean boost (i.e., a transformation of the form $x^i \mapsto x^i + v^i t$ for some constant vector $v^i$—note that this is a subset of the full group of Galilean transformations, which includes also rotations and translations), one finds that the Lagrangian changes by a total derivative:

36 $$\begin{aligned} \delta _G {\mathcal {L}} = \frac{d}{dt}\left( M x^i v^i \right) . \end{aligned}$$

Here, $\delta _G {\mathcal {L}}$ represents the variation of ${\mathcal {L}}$ under the Galilean boost. This variation of ${\mathcal {L}}$ is related to the Euler-Lagrange equations obtained by variation of the fields, and to the ‘naïve’ conserved charge ${\tilde{Q}}_G$ associated to Galilean boosts, in the usual way:^40^

37 $$\begin{aligned} \delta _G {\mathcal {L}} = \text {ELEs} + \frac{d}{dt} {\tilde{Q}}_G , \end{aligned}$$

On-shell and using our above result, we therefore find

38 $$\begin{aligned} \frac{d}{dt}\left( {\tilde{Q}}_G - M x^i v^i \right) = 0 , \end{aligned}$$

so that the ‘true’ conserved charge associated with Galilean boosts is $Q_G = {\tilde{Q}}_G - M x^i v^i$. Since ${\tilde{Q}}_G = p^i \delta _G x^i = M {\dot{x}}^i v^i t$, we have

39 $$\begin{aligned} Q_G = M {\dot{x}}^i v^i t - M x^i v^i. \end{aligned}$$

Taking now the Poisson bracket of $Q_G$ with the Noether charge $Q_P$ corresponding to infinitesimal translations $a^i$ gives

40 $$\begin{aligned} { \left\{ Q_G , Q_P \right\} }_{\text {PB}} = - M v^i a^i . \end{aligned}$$

On the right hand side, we see the mass generator *M*. Thus, the algebra of conserved charges is a representation of what we have called the Bargmann algebra. Since the Poisson brackets of conserved charges should mirror the Lie algebra structure of the associated symmetries,^41^ this in turn means that the coupling of a massive free particle to a Galilean background leads naturally to the central extension of the Galilean group. The mass gauge field $m_a$ is thus introduced when we ‘gauge’ the Bargmann algebra.^42^

## Footnotes

1 Actually, the situation here is delicate. In his discussion, Synge has in mind what we call below **NEP**: “that the effects of a gravitational field are indistinguishable from the effects of an observer’s acceleration” (Synge 1960, p. ix). We concur with Synge, insofar as this principle makes no sense in a geometrized theory of gravitation, such as general relativity. There are, however, other versions of the equivalence principle which we do regard as being contentful in general relativity: in this sense, we disagree with the spirit of Synge’s claim.

2 More precisely, what we mean here is this: ‘equivalence principle’ can be disambiguated in many different ways; some of the resulting principles hold within a theory, and can be deployed in order to better understand its conceptual architecture; other resulting principles hold between theories.

3 For the *locus classicus* on this theory, see (Malament 2012, ch. 4).

4 Recall that Newton’s Corollary VI reads as follows: “If bodies are moving in any way whatsoever with respect to one another and are urged by equal accelerative forces along parallel lines, they will all continue to move with respect to one another in the same way as they would if they were not acted on by those forces.” (Newton 2014, p. 99).

5 See (Aldrovandi and Pereira 2013) for an introduction to this theory, and (Aldrovandi et al. 2003) for some discussion of the equivalence principle in this theory complimentary to our own.

6 At this point, the following is stipulative, *à la* (Malament 2012, pp. 121–1, 252). There is a rich literature on deriving geodesic motion in theories such as general relativity: see e.g. (Geroch and Jang 1975; Geroch and Weatherall 2014; Tamir 2012).

7 Some authors choose to include explicitly $\xi ^a$ in the models of general relativity and Newton-Cartan theory; nothing significant (at least for our purposes) rides on this choice.

8 The case is thus distinguished from that of integrable Weyl geometries—cf. Almeida et al. 2014.

9 For other recent work on the equivalence principle, see (Brown and Read 2016; Di Casola et al. 2015; Nobili et al. 2013).

10 Our version of $\mathbf{WEP} _{\mathbf{1}}$ is very similar to that presented by Lehmkuhl, who in turn draws on the presentation of Will (2018, p. 16). Cf. also (Thorne et al. 1973, p. 3571).

11 We thank an anonymous referee for pushing us to be explicit about the conceptual difficulties encountered by $\mathbf{WEP} _{\mathbf{1}}$.

12 But not *vice versa*—see (Lehmkuhl 2021, p. 4).

13 See footnote 4. Note also that this will hold only locally in the relativistic context: if one considers a system of bodies at rest with respect to one another in special relativity, and then boosts to a Rindler frame (i.e., a uniformly accelerating frame), then one finds that all such bodies are not necessarily subject to the same inertial accelerations in that frame. Note also that this principle would be violated if different bodies were to couple to different affine connections, for this would lead to different connection coefficients appearing in their respective equations of motion when described in an accelerating frame. (Thus, we are close here to Trautman’s version of the equivalence principle: that there is a privileged affine connection (Trautman 1966).)

14 We have modified slightly Knox’s version of **NEP** to make explicit reference to a uniformly accelerating frame; our version is closest to that presented by Saunders (2013, p. 37).

15 Importantly, throughout this paper, we intend ‘relativistic context’ to include both general relativity and teleparallel gravity; likewise, we intend ‘Newtonian context’ to include both Newtonian gravity and Newton-Cartan theory. This qualification is important, for strictly speaking a principle such as **NEP** makes no sense within the framework of a geometrized theory such as general relativity: it only makes sense in the corresponding force theory. See our discussion of **EEP** below for further details.

16 See footnote 13.

17 The rocket experiment is sometimes referred to as ‘Einstein’s elevator’. There are natural questions regarding the parallels between the rocket and the earlier thought experiment of Galileo’s ship. In brief, the difference are as follows. Galileo’s ship is intended to demonstrate the relativity principle: there is a multiplicity of frames in which one’s description of a given physical scenario maximally simplifies, with those frames being related by uniform boosts. By contrast, Einstein’s rocket is intended to demonstrate an equivalence principle of the form **NEP**: modification of the original physical scenario to involve an extra force (*viz*., that associated with a uniform gravitational field) is indistinguishable from describing the original physical scenario in a non-inertial, accelerating frame of reference.

18 It is illuminating to contrast this conceptual unification on the part of Einstein (for more on which see Lehmkuhl 2014) with his earlier unification of compensating Lorentz-Fitzgerald contraction effects is his 1905 presentation of special relativity (Einstein 1905).

19 Cf. (Aldrovandi et al. 2003, p. 546).

20 Since in this paper we are dealing with pointwise constructions, we do not consider translations, hence our focus on the Lorentz rather than the Poincaré group. It is worth noting, though, that there are various ways of making sense of the notion of ‘local Poincaré symmetries’ which we endorse—for discussion, see (Brown 1997; Fletcher 2020; Read et al. 2018).

21 Cf. again Trautman’s version of the equivalence principle, introduced in footnote 13.

22 One could say: our concern in this paper is with zero-dimensional observers—in which case, such curvature terms cannot have any practical effect. Cf. (Aldrovandi et al. 2003).

23 Here is how she puts the principle: “To any required degree of approximation, given a sufficiently small spatial region, it is possible to find a freely falling reference frame with respect to whose associated coordinates the motions of bodies amongst themselves are indistinguishable from those expected in the absence of external forces” (Knox 2014, p. 874).

24 From the point of view of Knox exegesis, Knox takes the arbitrariness of the gravity-inertia split encapsulated in  as *prima facie* evidence—assuming that symmetry-related solutions of a given theory should be regarded as representing the same physical states of affairs (cf. Møller-Nielsen 2018)—for **EEP**, and in turn for $\mathbf{SEP}_{{\mathbf{1, EEP}}}$.

25 The concept of a normal frame can be generalized to the case of torsionful spacetimes by using non-holonomic coordinates (Aldrovandi et al. 2003; Iliev 1998). See (Knox 2013, §4.2) for some philosophical discussion of the physical content of such coordinate systems.

26 It has been put to us that teleparallel gravity is not empirically equivalent to general relativity, for (the claim goes) it has more gravitational wave polarizations. But this is false: see e.g. (Bambaa et al. 2013).

27 Here we have already set the spin connection to vanish, so are dealing with ‘pure tetrad’ teleparallel gravity. If we do not set the spin connection to vanish, (12) takes a more general form; in that case, we are dealing with ‘covariant’ teleparallel gravity. Note that covariant teleparallel gravity is the now the preferred form of the theory, for it does not face issues of consistency when coupling to spinorial matter. For more on this, see e.g. (Kopczyński 1982; Leclerc 2005).

28 Here, $\partial$ is a coordinate derivative operator, in the sense of (Malament 2012, pp. 64–65).

29 For some related discussion of force theories equivalent to general relativity, see (Weatherall and Manchak 2014); for critical engagement with that piece (with which we concur), see (Dürr 2021).

30 On the other hand, readers seeking further technical details are referred to the original source in the physics literature (Read and Teh 2018).

31 See (Andringa et al. 2011) for a clear introduction to this formalism.

32 For more on the physical motivations for introducing this object, see the appendix.

33 Recall that in order to identify elements of a fibre associated with a point $p \in M$ with elements of a fibre associated with a point $q \in M$ infinitesimally separated from *p*, one must use a spin connection, , related to the spacetime connection on the base manifold via the ‘vielbein hypothesis’, . (Here, we have used four-dimensional indices, for illustrative purposes.)

34 See (Bekaert and Morand 2016) for a derivation of this result.

35 For convenience here and at some points below, we here make use of differential forms notation.

36 Though see (Dewar 2019; Martens & Read 2021) for some discussion on this point.

37 The conceptual status of these links is, therefore, different from those relating $\mathbf{WEP} _{\mathbf{1}}$, $\mathbf{WEP} _{\mathbf{2}}$ and **NEP**, for the former link versions of the equivalence principle in different theories; the latter link versions of the equivalence principle within one (degeometrized) theory.

38 See (Read and Teh 2018), in which this was shown for the case of ‘null reduction’, where a Newton-Cartan model is embedded directly into a (higher-dimensional) model of general relativity. It would be worthwhile (and, we expect, straightforward) to demonstrate that the same commuting diagram can be constructed when one takes the Newtonian limit using frame theory.
